# Integrative analysis of copy number and gene expression in breast cancer using formalin-fixed paraffin-embedded core biopsy tissue: a feasibility study

**DOI:** 10.1186/s12864-017-3867-3

**Published:** 2017-07-11

**Authors:** Mahesh Iddawela, Oscar Rueda, Jenny Eremin, Oleg Eremin, Jed Cowley, Helena M. Earl, Carlos Caldas

**Affiliations:** 1 Cancer Research UK Cambridge Institute, Li Ka Shing Centre, Robinson Way, Cambridge, CB2 0RE UK; 2 Department of Oncology, University of Cambridge, Addenbrooke’s Hospital, Hills Road, Cambridge, UK; 3 Cambridge Breast Unit, Addenbrooke’s Hospital, Cambridge University Hospitals NHS Foundation Trust, NIHR Cambridge Biomedical Research Centre and Cambridge Experimental Cancer Medicine Centre, Cambridge, UK; 4 Department of Anatomy & Developmental Biology, Monash University, Clayton, VIC 3800 Australia; 5 School of Clinical Sciences, Monash University, Clayton, Australia; 6 Research and Development, Lincoln Breast Unit, Lincoln County Hospital, Lincoln, UK; 7 Nottingham Digestive Disease Centre, Faculty of Medicine and Health Sciences, University of Nottingham, Queen’s Medical Centre, Nottingham, UK; 8 PathLinks, Lincoln County Hospital, Lincoln, UK

**Keywords:** Gene Expression, Copy Number, FFPE, Genomics, Breast Cancer, DASL, Molecular Inversion Probes Assay, Oncoscan Array, Molecular Markers

## Abstract

**Background:**

An absence of reliable molecular markers has hampered individualised breast cancer treatments, and a major limitation for translational research is the lack of fresh tissue. There are, however, abundant banks of formalin-fixed paraffin-embedded (FFPE) tissue. This study evaluated two platforms available for the analysis of DNA copy number and gene expression using FFPE samples.

**Methods:**

The cDNA-mediated annealing, selection, extension, and ligation assay (DASL™) has been developed for gene expression analysis and the Molecular Inversion Probes assay (Oncoscan™), were used for copy number analysis using FFPE tissues. Gene expression and copy number were evaluated in core-biopsy samples from patients with breast cancer undergoing neoadjuvant chemotherapy (NAC).

**Results:**

Forty-three core-biopsies were evaluated and characteristic copy number changes in breast cancers, gains in 1q, 8q, 11q, 17q and 20q and losses in 6q, 8p, 13q and 16q, were confirmed. Regions that frequently exhibited gains in tumours showing a pathological complete response (pCR) to NAC were 1q *(55%)*, 8q *(40%)* and 17q *(40%),* whereas 11q11 (*37%*) gain was the most frequent change in non-pCR tumours. Gains associated with poor survival were 11q13 *(62%)*, 8q24 *(54%)* and 20q *(47%)*. Gene expression assessed by DASL correlated with immunohistochemistry (IHC) analysis for oestrogen receptor (ER) [area under the curve (AUC) = 0.95], progesterone receptor (PR)(AUC = 0.90) and human epidermal growth factor type-2 receptor (HER-2) (AUC = 0.96). Differential expression analysis between ER+ and ER– cancers identified over-expression of *TTF1, LAF-4 and C-MYB* (*p* ≤ 0.05), and between pCR vs non-pCRs, over-expression of *CXCL9, AREG, B-MYB* and under-expression of *ABCG2*.

**Conclusion:**

This study was an integrative analysis of copy number and gene expression using FFPE core biopsies and showed that molecular marker data from FFPE tissues were consistent with those in previous studies using fresh-frozen samples. FFPE tissue can provide reliable information and will be a useful tool in molecular marker studies.

**Trial Registration:**

Trial registration number ISRCTN09184069 and registered retrospectively on 02/06/2010.

**Electronic supplementary material:**

The online version of this article (doi:10.1186/s12864-017-3867-3) contains supplementary material, which is available to authorized users.

## Background

Breast cancer remains a major public health problem in the western world, with a significant impact on mortality and morbidity. Despite the considerable amount of research that has been carried out, oestrogen receptor (ER), progesterone receptor (PR) and human epidermal growth factor receptor 2 (HER-2) remain the only routinely used molecular markers in breast cancer [1, 2]. Multiplex markers are being introduced for particular clinical situation but these are currently expensive and not universally helpful [3, 4]. Breast cancer is a heterogeneous disease and large numbers of samples are needed to reliably characterise different subtypes with confidence [5, 6]. Unfortunately, availability of FF (fresh frozen) tissues for translational research are limited. However, on the other hand, abundant supplies of FFPE (formalin-fixed paraffin-embedded) tissues are readily available for use, provided suitable assays are in place [7] .

One of the main problems associated with FFPE samples is that cellular RNA is degraded and therefore the platforms must be adapted for analysis; this is one of the challenges of using FFPE samples. Standard microarrays used for FF samples are not suitable for analysis of FFPE samples because conventional in-vitro transcription is not amenable to analysis of degraded samples [8].

Two main technologies are employed for gene expression profiling using FFPE tissue: expression microarrays using oligo-(dT) priming (Affymetrix and other custom-made microarrays) and a combination of oligo-(dT)/random hexamers together with gene-specific primers utilised by the cDNA-mediated annealing, selection, extension and ligation (DASL) assay [9, 10]. In the DASL assay, the expression of 502 genes is assessed, each using three primers. Only a 40 bp sequence is required to determine mRNA abundance, and hence this method is generally suitable for the analysis of degraded FFPE samples [11]. Both of these platforms have been used for gene expression profiling of FFPE core biopsy tissues. Bibikova et al. showed that the DASL platform can be used to assess gene expression profiles and evaluate differentially-expressed genes in cancerous and normal tissues [11]. Furthermore, we have shown reliable expression profiles can be generated using FFPE tissue with profiles overlapping with those from FF tissues [12]. DASL has been used to generate signatures of molecular markers associated with poor outcome in prostate cancer [13], genes associated with poor outcome in melanoma [14], and in oesophageal cancer [15].

Copy number alterations may have profound effects on cancer development, and on progression and response to treatment. Thus, characterisation of pivotal changes and their molecular pathways in breast cancer may have important clinical implications. Several well-defined copy number alterations have been identified in cancer, such as *ERBB2* in breast cancer [16], *N-MYC* in neuroblastoma [17, 18] and *EGFR-1* in head and neck tumours and gliomas [19–21], which have prognostic as well as predictive implications.

Until recently, there has been uncertainty about the value of FFPE tissue in clinical prognostic marker studies because of technical problems such as difficulties in DNA extraction, low quantities of extractable DNA, and the problems associated with using such DNA owing to degradation. Technological advances in DNA extraction protocols and platforms used for genome-wide copy number analysis (CNA) using DNA extracted from FFPE tissue have progressed rapidly over the past few years [22, 23]. Newer platforms used for CNA require less DNA, rendering analysis of FFPE material a more realistic and reliable technique.

The Molecular Inversion Probe (MIP) assay is a very promising platform for CNA in both FF and FFPE material [24, 25]. Degraded DNA can be used in this assay as it only requires a 40-bp sequence for CNA, making it suitable for DNA analysis of FFPE material. This technique has been used to define new copy number changes in Ewing’s sarcomas and in childhood leukaemias [26, 27]. The reliability of this method has also been assessed using samples obtained from several institutions. These data suggest that this technology will be a useful tool for CNA using FFPE tissue, and the low DNA requirement renders it ideal for analysis of core biopsies of samples or in situations where only limited material is available.

Integrative analysis of both copy number and gene expression has been shown to be a valuable method for identifying new molecular markers in cancer. In one of the largest studies in breast cancer, Curtis et al. showed that breast cancer can be divided into 10 subtypes using integrative analysis [5]. Chin et al. further showed that novel ER-negative breast cancers can be identified using analysis of gene expression and copy number [28]. If such analysis can be carried out on FFPE tissue, it will enable the evaluation of larger numbers of clinical samples and so lead to faster progress in translational research [29]. This potential has a role in the discovery, as well as the validation, of potential prognostic markers, and as the samples are readily available it is a useful source of material for translational research.

The previous study looked a the role of FFPE analysis using the whole genome DASL assay and its role in gene expression profiling of fresh and FFPE tissue [12].

In this study, profiling of copy number changes and gene expression was carried out using the same FFPE clinical samples. Routinely collected core biopsies were used, to evaluate whether these assays can be used for molecular marker studies. This study used the DASL Cancer Panel Assay to evaluate the FFPE samples. To the best of our knowledge this is the first genome-wide copy number and expression profiling study using FFPE core biopsies from patients with large breast cancers undergoing neoadjuvant chemotherapy (NAC).

## Methods

### Samples

In this study, 63 samples from patients with large locally advanced breast cancer (LLABC) (T2 > 3 cm, T3 or 4, N1,2) treated with NAC trial were used. The trial to evaluate the safety and efficacy of weekly vs. 3 weekly docetaxel (33.3 mg/m^2^ or 100 mg/m^2^) administration after 3 weekly Adriamycin (60 mg/m^2^) and cyclophosphamide (100 mg/m^2^) for patients diagnosed with LLABC in the period 1999–2002. All patients underwent surgery 4 weeks post NAC, and a core biopsy was performed in all patients prior to starting treatment and patients consented to enter the neoadjuvant study. Following ethical approval, samples from these patients were used for gene expression profiling and CNA. Matched normal and tumour tissue taken at the time of surgery were also used in this cohort.

The copy number analysis was undertaken using both pre-NAC and normal tissues while gene expression was only undertaken with pre-NAC samples only. The study used core biopsy tumour tissue to evaluate these platforms as this is the most readily available sample. Expression analysis was undertaken using multiple replicates. While for copy number analysis, both normal and cancer tissues were used. The samples were evaluated for ER, PR and HER-2 expression using IHC. The ER and PR were reported as positive and negative and HER-2 expression reported as 0 & 1(no expression), 2 (equivocal) and 3 (over-expressed).

### DNA extraction

Ten 10 μm sections from FFPE blocks were deparaffinised with xylene, and DNA was extracted using the Qiagen DNeasy Tissue kit (Qiagen^®^) according to the manufacturer’s protocol with several modifications. The modifications included an initial incubation at 95 °C for 15 min (mins) in RTL buffer, followed by 5 mins. at room temperature, before proteinase K treatment was performed. Next, proteinase K was added every 24 h and digested for 72 h at 56 °C in a thermomixer. The extracted DNA was quantified using UV spectroscopy at 260 nm.

### RNA extraction

Between five and eight 5-μm sections were cut from each block of FFPE tissue, and then the samples were deparaffinised with xylene and proteinase K treated for 14 h. Purification and DNase treatment – were performed using a Roche High Pure RNA Purification Kit (Roche Applied Sciences) and total RNA was stored at −80 °C after extraction.

### DNA and RNA quality control

The amount of DNA extracted was quantified using the NanoDrop and PicoGreen assay. RNA was quantified using the NanoDrop system, and the extent of degradation was measured using RT-qPCR for *RPL13A* mRNA with the primers defined by Illumina [7].

### cDNA-mediated annealing, selection and ligation (DASL) assay and gene expression profiling

During the DASL assay, total RNA was converted to cDNA in a reverse transcription reaction using biotinylated oligo-(dT) _18_ and random hexamers. Pairs of query oligonucleotides, with three unique pairs for each of 502 genes, were annealed to complementary sequences (~50 base pairs) flanking the specific cDNA target site. The biotinylated cDNA was then bound to streptavidin beads, and mis-hybridised and non-hybridised oligonucleotides were washed away. Through a primer extension and ligation process, the biotinylated product was generated, and this was then amplified by universal fluorescent primers using conditions described below to fluorescently label and amplify the template cDNA. The 5′ primers were labelled with Cy3 and Cy5 fluorogenic dyes, respectively, while the 3′ primer contained the address sequence that is complementary to a secondary address sequence located on array (SAM). The amplified cDNA was then denatured and hybridised to the SAM at 60 °C in a hybridisation oven with an oscillating table. Following overnight hybridisation, the array was washed and then scanned using the BeadArray reader (Illumina™). Image processing and intensity data files were analysed using BeadStudio software.

The probes used for the DASL assay were sourced from a cancer panel which consisted of 502 genes generated using 10 publically available data sets (http://www.gtbiotech.com.tw/pdf/DASL%20Assay%20Work%20Flow.pdf). The selection was based on their frequency of citation in the lists and also their association with cancer.

### Allelic composition analysis: molecular inversion probe (MIP) assay

DNA (2.35 μl) was mixed with 1.1 μl of 53 K probe pool (200 amol/μl/probe) and placed in a 96-well plate in ice. The reaction mixture was incubated at 20 °C for 4 mins, and 95 °C for 5 mins, then 58 °C overnight. Next, 13 ml of a second enzyme mix and buffer were added. The MIPs were circularised by the addition of 4 μl of dinucleotides (dATP with dTTP, dCTP and dGTP) and mixed at 58 °C for 10 mins. The non-circularised probes and genomic DNA were eliminated by the addition of 4 μl of exonuclease mix and incubated at 37 °C for 15 mins, and then heat treated. The circularised probes were linearised by adding cleavage enzyme mix at 37 °C for 15 mins, then subjected to universal primer amplification for 18 cycles at 95 °C for 20 s, 64 °C for 40 s, and 72 °C for 10 s. For the labelling reaction, products were further amplified with labelled primers for 10 ten cycles, and then subjected to cleavage by a digestion enzyme mix at 37 °C for 2 h. The products were mixed with a hybridisation cocktail, denatured and hybridised to Affymetrix Universal 70 K Tag arrays at 39 °C for 16 h (two arrays per sample). After overnight hybridisation, arrays were washed on Affymetrix GeneChip Fluidics Station 450 and stained by streptavidin-phycoerythrin (SAPE) at 5 mg/ml (Invitrogen).

### Data analysis

The data from the MIP arrays were normalized using a 2pSE structuring element, which has been established as the best method for normalising MIP data [30]. Two different algorithms were used for copy number analysis. First, the data was then segmented using the circular binary segmentation (CBS) algorithm [31]. This algorithm splits the data into segments of equal copy number. A more refined analysis was done using the reverse jump array comparative genomic hybridisation (RJaCGH) algorithm, that fits a non-homogeneous hidden Markov model using reversible jump Markov chain Monte Carlo computation, and that takes into account model uncertainty using Bayesian model averaging [32, 33]. This method estimates the probability that a region/gene has a copy number alteration (rather than a *p* value or smoothed mean) and is useful in both basic and clinical applications. This algorithm also takes into account the distance between the probes, and is for this platform where there is an uneven distribution of markers on each chromosome. For example, in densely covered areas, the copy number of one probe is indicative of the copy number throughout the whole area. In contrast, in poorly covered areas where the probes are further apart, it is possible that copy number alterations will have occurred, but not be detected, so each probe provides much less information about the state of the neighbouring probes. Therefore, the distance between the probes needs careful consideration to ensure that the data provided by the consecutive probes was recorded accurately. This algorithm has the ability to assess data for each chromosome or on a genome–wide basis.

Agglomerative hierarchical clustering was carried out using the base R package function hclust with Ward’s method. This allows compact clusters to be generated from the data and uses an analysis of variance approach to determine the distance between the clusters [34]. The smaller the increase in the sum of the within-group sum of squares, when two clusters are merged, the closer the two clusters. The within-group sum of squares was defined as the sum of squares of the distance between all objects in the cluster and the mean centroid of the cluster.

Robustness of the cluster was assessed using the R package pvclust method [35]. This method assesses the uncertainty in hierarchical clustering by calculating quantiles called a *p*-value via multi-scale bootstrap resampling. Gene ontology assessment was carried out for the data using the Bioconductor package GOstats [36].

Quality control checks were carried out on the extracted data to identify any spatial effects or problems with probes. For the initial analysis, the data were quantile normalised, and hierarchical clustering was undertaken to assess the robustness of the platform. Hierarchical clustering is a robust method used to assess the relationship between samples. To assess robustness, a more detailed analysis of the probes and replicate arrays was carried out with the aim of further removing any noise. The MA and XY plots for the red and green channels were used to assess the array quality between the replicates, and those that did not agree with the replicates were removed.

For gene expression profiling, the majority of samples were assessed with multiple replicates. Pearson’s correlation coefficient (r) was used to assess the reproducibility of the probes between replicates, and where r^2^ < 0.7 these data were excluded. The genes involved were initially selected as they passed the stringent criteria described. The average red and green signals of these probes were calculated and the data were quantile normalised. A subsequent analysis of the data was then undertaken.

## Results

### Patient characteristics

Forty-three of the 63 (68%) patients treated in the NAC had sufficient DNA following extraction for analysis, while RNA was extracted from 52 of 63 (82%) FFPE core biopsies. For CNA, 43 samples were used, while 46 samples were used for gene expression analysis. Clinical information and the tumour characteristics of the patients in this cohort are shown in Table 1 (Additional file 1). The mean age of the patients was 50 years (range 32–70), 33 (72%) of the tumours were T2, while 27 (58%) were grade 3. Of the patients, 30 (65%) had ER positive and a pCR was found in 30% (14/46) of the patients following NAC.

**Table 1:** Characteristics of the patients used for expression and copy number analysis

Mean Age (Years)	50
Range	32–70
Tumour Type	
Ductal Carcinoma	44 (96%)
Lobular Carcinoma	2 (4%)
Tumour Grade	
1	3 (7%)
2	16 (35%)
3	27 (58%)
Tumour Stage (Breast)	
T1	5 (11%)
T2	33 (72%)
T3	6 (13%)
T4	2 (4%)
Oestrogen Receptor	
Positive	30 (65%)
Negative	16 (35%)
Pathological Response	
Pathological Complete Response (pCR)	14 (30%)
No Pathological Complete Response (Non-pCR)	32 (70%)

### Correlation between IHC, gene expression and copy numbers

The median DNA yield per sample was 325 ng (range 198–2000) while the RNA yield was 1800 ng (range 114–3696). The matched normal tissues from a cohort of samples were used for normalising copy number data. Six of the 43 tumours exhibited gains or amplification in *HER-2* when assessed using the MIP assay (14%). When correlated with assessment by immunohistochemistry (IHC), five samples had an IHC score of 3+ (83%), a further three samples had an IHC score of 2 + .

Gene expression, as assessed by the DASL assay, was compared with IHC to assess the reliability of the platform. The IHC scores for ER, PR, HER-2 and BCL2 proteins were compared with DASL gene expression results. Gene expression was dichotomised using a median cut-off, and an area under the curve (AUC) was generated to compare the two platforms (Table 2).

**Table 2:** The AUC for each DASL probe compared with IHC and the correlation between gene expression assessed using DASL and IHC score (*statistically significant *p* < 0.05, **p < 0.0001)

Marker	AUC for each probe			Correlation for each probe		
	1	2	3	1	2	3
Oestrogen receptor	0.95**	0.83*	0.75*	0.77**	0.57**	0.44*
Epidermal growth factor receptor −2	0.96**	0.96**	0.94**	0.75**	0.75**	0.81**
Progesterone receptor	0.72*	0.90*	0.90*	0.36*	0.66*	0.64*
BCL2 expression	0.74*	0.8*	0.46	0.40*	0.60*	0.11

**Fig. 1:**
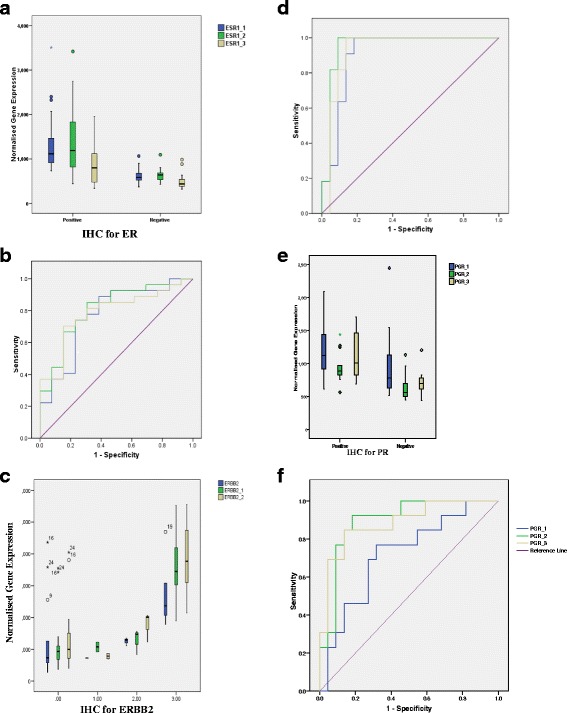
Box plot showing gene expression in cancers according to IHC status and the Area Under the Curve (AUC) for each of the probes for each marker (number of samples). **a** & **b** = oestrogen receptor expression (positive = 30 & negative = 16), (**c** & **d**) = ERBB-2 expression 0 (15), 1 (2),2 (5) & 3 (6), (**e** & **f**) = progesterone receptor expression (positive- 20 & negative 20) (Outlier samples shown by stars and numbers)

Gene expression of *ESR1* was assessed using the three probes of the DASL assay, compared with the IHC ER-positive and -negative status (Fig. 1). As shown, ER-positive tumours exhibited elevated *ESR1* expression compared with ER-negative cancers, and the difference was statistically significant (linear regression *p* value = 0.023). The AUC analysis comparing the two platforms exhibited robust overlap with values of 0.77–0.8 (*p* = 0.002). However, the correlation between the two platforms was in the range of 0.4–0.8, depending on the probes used. The probe-specific variation in the signal is very clear as the *ESR1_3* had a lower signal compared with the other two probes and, as shown, this also has a lower AUC. Detailed analysis of the primer binding sites on the *ESR1* gene showed that the probe with a lower AUC binds to a site closer to the 5′-UTR (un-translated region) than the other two probes, with a high gene expression signal (Additional file 2).

A similar comparison was carried out for *HER-2*, where a significant difference in gene expression was found according to the HER-2 status assessed by IHC, with the cancers classed as IHC 3+ having a greater expression value (*p* = 0.022, Fig. 1). The AUC was 0.91–0.95 using this probe (*p* < 0.001) and the correlation was 0.75–0.81. When this analysis was used to assess *PGR* and *BCL2* status, there was again a difference in gene expression between those classed as positive and those classed as negative (linear regression *p* = 0.0002 for *PGR* and *p* = 0.01 for *BCL2*). The AUC for these two genes was >0.70 (*p* < 0.05) for all probes except for one of the *BCL2* probes, and the correlation was also significant for all probes except this *BCL2* probe (Table 2).

### Copy number changes in breast cancer

**Fig. 2:**
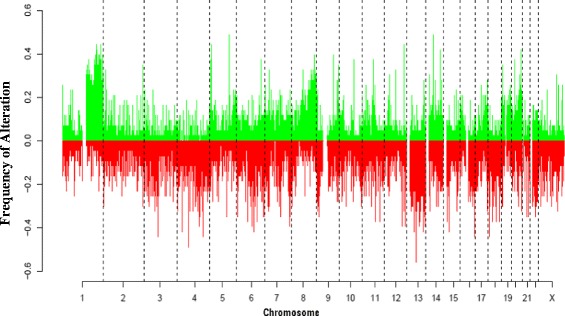
Chromosomal plot showing copy number aberrations in all tumours analysed in the study (X-axis- chromosome number 1-X & Y axis frequency of alteration, Gains-green and Losses-red)

The regions with the highest frequency of gains (>30%) comprised 1q (42%), 8q (40%), 11q (40%), 17q (32%) and 20q (35%) (Fig. 2). A high frequency of gains was also observed in smaller regions of 5p15.31 (45%), 5q15.2 (51%), 5q23.1 (53%), 12q24.22 (49%) and 14q21.3 (51%). The regions identified as having the highest frequency of loss comprised 4p (34%), 4q (34%), 6q (43%), 8p (30%), 13q (30%), 16q (30%) and 18q (35%) (Fig. 2) (Additional file 3).

The copy number data were analysed using clustering methods to identify any robust groups in this cohort. Unsupervised clustering showed that there were two distinct groups – 1 and 2 – with distinct pathological features. In Group 1, 83% of the cancers (15/18) were ER-positive tumours. Group 2 was more heterogeneous, with 10 ER-positive and 9 ER-negative tumours (Chi squared test, *p* value = 0.001). The robustness of the clusters was assessed using the pv-clust software package, which provides an approximate unbiased *p* value and bootstrapping probability. This showed that the clusters are distinct with defined features.

The effect of copy number gains of known oncogenes such as *ERBB2, CCND1* and *C-MYC* in breast cancer, was used to assess the robustness of the platform. Of the breast cancers assessed, there were gains in *HER-2* in 33%, *CCND1* in 32%, and *C-MYC* in 39%. The survival analysis of the patients with these gains showed that *HER-2* amplification and *C-MYC* gain was associated with poor overall survival and that the frequency of these gene CNA are similar to that described in other breast cancer studies.

### Description of gains and losses and correlation with pathology

The ER-positive tumours had the highest frequency of gains in 1q and 8q, while there were focal gains in 11q11 and 20q13. In ER-negative tumours, 11p and 17q had the highest frequency of gain.

When grade 3 and 1 tumours were compared, 8q23.3–q24.11 (49% vs 17%), 8q24.11 (48% vs 16%) and 8q24.21 (43% vs 9%) were found to be more common in grade 3 lesions, while gains in 1q21.1–q21.2 (55% vs 29%), 1q24.1 (54% vs 25%), 1q21.2–21.3 (54% vs 26%), and 1q22 (55% vs 27%) were found to be more common in grade 1 tumours. These changes were statistically significant following multiple testing (FDR < 0.05). Loss at 16q was seen in 40% of the grade 1 tumours compared with 18% of the grade 3 tumours.

When CNA and responses to NAC were assessed, gains were frequent in 1q (55%), 8q (40%) and 17q (40%) among pCR tumours, while 11q11 gain was more frequent among the non-pCR tumours. The data also showed that 8p (40%) and 13q (70%) losses were more common among the pCR tumours, but none of these regions were statistically significant.

Common regions of alteration associated with poor overall survival comprised gains in 11q13 (62%), 8q24.21 (54%), 8q24.22 (54%) and 20q13.13 (47%) (*p* < 0.1). The region most frequently gained in patients with a good overall survival was 1q. There were several regions of 1q and these were: 1q31, 3 (39%), 1q32.2 (39%), 1q41 (39%) and 1q31 (39%).

### Gene expression profiling of the pre-treatment core biopsies – differential expression analysis

Differential gene expression analysis was carried out to assess overlap with published data, and which would act as a validation of the role of the assay. Differentially expressed genes between the 30 ER-positive and 16 ER-negative cancers were assessed and, as shown, all the three *ESR1* probes were classified as highly expressed in ER-positive cancers and were significant on multiple testing (FDR *p* ≤ 0.001) (Table 3) (Additional files 4, 5, 6, 7).

**Table 3:** Top 10 differentially expressed genes (each probe) between oestrogen receptor positive and negative cancers

FDR	Log-Fold Change	Genes	Gene name
1.90E-07	1.992	**TFF1**	TREFOIL FACTOR 1 (BREAST CANCER, OESTROGEN-INDUCIBLE SEQUENCE EXPRESSED IN)
7.13E-06	1.511	**ESR1**	OESTROGEN RECEPTOR 1
6.90E-05	1.568	**ESR1**	OESTROGEN RECEPTOR 1
1.00E-03	1.012	**LAF4**	NUCLEAR PROTEIN RELATED TO AF4
1.00E-03	1.299	**ESR1**	OESTROGEN RECEPTOR 1
2.00E-03	0.780	**C-MYB**	V-MYB MYELOBLASTOSIS VIRAL ONCOGENE HOMOLOGUE (AVIAN)
3.00E-03	1.464	**AREG**	AMPHIREGULIN (SCHWANNOMA-DERIVED GROWTH FACTOR)
3.00E-02	−0.964	CD44	CD44 ANTIGEN (INDIAN BLOOD GROUP)
3.00E-02	0.765	**LAF4**	NUCLEAR PROTEIN RELATED TO AF4
3.50E-02	0.800	ERBB4	V-ERB-A ERYTHROBLASTIC LEUKEMIA VIRAL ONCOGENE HOMOLOGUE 4 (AVIAN)

Well-characterised ER regulated genes are shown in bold and note each ESR1

This analysis also showed that other oestrogen-regulated genes such as *TFF1, PGR* and *C-MYB* were also present in the top 25 genes classified as highly differentially expressed (FDR *p* < 0.05). *LAF4* and *AREG* showed high expression in ER-positive breast cancers, and all three transcripts showed high expression among ER-positive breast cancers. Due to small the sample size these changes could be biologically not significant and need further validation.

Grade 1 and 3 tumours were also assessed to identify those genes that were differentially expressed between the high-grade and low-grade tumours. As shown below, *BIRC5, ERBB4, SOD1, ARHGDIB* and *CDC25B* were the top 4 differentially expressed genes (Table 4). All the genes, apart from *ERBB4*, were over-expressed in grade 3 tumours compared with grade 1 tumours, and all three *ERBB4* transcripts were present in the top 25 genes.

**Table 4:** Table showing the top 10 differentially expressed genes (each probe) between grade 1 and grade 3 tumours

FDR	Log-Fold Change	Genes	Gene name
0.186	0.271	BIRC5	BACULOVIRAL IAP REPEAT-CONTAINING 5 (SURVIVIN)
0.186	−0.337	ERBB4	V-ERB-A ERYTHROBLASTIC LEUKAEMIA VIRAL ONCOGENE HOMOLOGUE 4 (AVIAN)
0.216	1.149	SOD1	SUPEROXIDE DISMUTASE 1, SOLUBLE (AMYOTROPHIC LATERAL SCLEROSIS 1 (ADULT))
0.247	0.723	ARHGDIB	RHO GDP DISSOCIATION INHIBITOR (GDI) BETA
0.247	0.622	CDC25B	CELL DIVISION CYCLE 25B
0.247	0.417	CXCL9	CHEMOKINE (C-X-C MOTIF) LIGAND 9
0.247	0.964	GRB7	GROWTH FACTOR RECEPTOR-BOUND PROTEIN 7
0.247	1.156	HIF1A	HYPOXIA-INDUCIBLE FACTOR 1, ALPHA SUBUNIT (BASIC HELIX-LOOP-HELIX TRANSCRIPTION FACTOR)
0.247	1.117	B-MYB	V-MYB MYELOBLASTOSIS VIRAL ONCOGENE HOMOLOGUE (AVIAN)-LIKE 2
0.247	−0.348	PGR	PROGESTERONE RECEPTOR

Although gene expression changes are statistically significant, some of the log fold changes are small and might not contribute to meaningful biological or physiological changes. This is especially the case when limited number of samples are used, hence both further validation and functional analysis is important for confirming the role of any potential markers in any study such as this.

The pCR rate following NAC study was 26% (12/46) and the expression profiles of these samples were used to assess differentially expressed genes between patients whose tumours had a pCR or non-pCR with NAC. The top three differentially expressed genes were *CXCL9, ARHA* and *ARHGDIB*, but this observation was not statistically significant on multiple testing (*p* < 0.11, Table 5). All three *ARHGDIB* alternate transcripts exhibited elevated expression in patients with pCR. There was lower expression of the multi-drug resistant transporter, *ABCG2* gene, in those tumours with a pCR following NAC.

**Table 5:** Table showing differentially expressed genes (each probe) between pathological complete responders (pCR) and those with no pathological complete response (non-pCR)

FDR	Log-Fold Change	Genes	Gene name
0.087	0.911	CXCL9	CHEMOKINE (C-X-C MOTIF) LIGAND 9
0.104	0.586	ARHA	RAS HOMOLOGUE GENE FAMILY, MEMBER A
0.104	0.700	ARHGDIB	RHO GDP DISSOCIATION INHIBITOR (GDI) BETA
0.104	0.925	BCL3	B-CELL CLL/LYMPHOMA 3
0.104	0.665	CD44	CD44 ANTIGEN (INDIAN BLOOD GROUP)
0.104	0.912	CDKN1B	CYCLIN-DEPENDENT KINASE INHIBITOR 1B (P27, KIP1)
0.104	0.638	CSF1R	COLONY STIMULATING FACTOR 1 RECEPTOR, FORMERLY MCDONOUGH FELINE SARCOMA VIRAL (V-FMS) ONCOGENE HOMOLOGUE
0.104	0.742	DCN	DECORIN
0.104	0.783	EGR1	EARLY GROWTH RESPONSE 1
0.104	0.600	LCK	LYMPHOCYTE-SPECIFIC PROTEIN TYROSINE KINASE

Gene ontology assessment of the top 50 differentially over-expressed genes indicated that the two most significantly-enriched GO categories were regulators of biological processes (61% of which were genes such as *MYB, ARHDIB and CDK9)* and cellular metabolic processes (61% of which were genes such as *MAPK14, SKIL and FGFR3*).

### Gene expression and copy number changes

The best performing 308 probes were correlated with copy number data. Overall, when all the reliable probe expression and copy number were compared, 25% probes had a Pearson correlation >0.26 and 50% more than >0.14. For certain probes, such as those targeting *ERBB2, CCND1, GRB7*, and *BIRC5*, there was good concordance observed between gene expression and locus copy number (Table 6). Nevertheless, in some instances, there was diminished correlation between detected copy number and quantified mRNA expression, as seen with probes 1 and 2 for the *FGF3* gene (*FGF-P1* and *FGF-P3*). Results for the well-characterised tumour suppressor gene *CDH1* indicated a correlation of 0.4, while *TOP2A* had a correlation of 0.44. A strong correlation was also found between copy number and mRNA expression for other genes in regions such as 11q13 (*CCD1*), 20q13 (*B-MYB*) and 17q12 (*ERBB2*).

**Table 6:** Correlation between gene expression and copy number for the robust probes used in the DASL assay (P1–3-identity of the probes used to assess each gene in the DASL assay) (Correlation between copy number and gene expression: 25% of probes >0.26 and 50% probes >0.14)

Gene name and DASL probe number	Correlation between expression and copy number	95% Confidence Interval
ESR1-P2	0.483	[0.161–0.712]
JUND-P1	0.490	[0.06–0.766]
MUC1-P2	0.497	[0.144–0.738]
BIRC5-P1	0.500	[0.17–0.729]
RAN-P1	0.503	[0.152–0.741]
RELA-P1	0.525	[0.172–0.758]
BIRC5-P2	0.527	[0.205–0.745]
COMT-P1	0.556	[−0.026–0.857]
FGFR1-P1	0.575	[0.283–0.769]
GRB7-P1	0.585	[0.192–0.816]
ERBB2-P1	0.733	[0.43–0.888]
CCND1-P1	0.773	[0.512–0.903]
HER-2-P2	0.800	[0.553–0.918]

Genes associated with a pCR and their relationship between copy number and gene expression were evaluated, *TNFAIP1* was revealed as a locus that demonstrated a very high correlation between copy number and expression (*r* = 0.99) (Table 7). None of the genes associated with pCR were statistically significant. Of the other genes, two of the three *HER-2* transcripts exhibited strong correlations between expression and copy number and were further associated with pCR. The genes associated with ER-negative status and with high copy number and gene expression ratios comprised *ERBB2, GRB2, STAT3, CCND1, MUC1* and *FGFR3*.

**Table 7:** Genes where there is a high correlation between copy number, expression and clinical feature

Clinical characteristic	Genes where copy number and expression correlation >0.75
Pathological complete response	*TNFAIP1, JUND, HER-2, NFKBIA, PDGF, IL13, CCND1*
Genes associated with ER-negative status	*GRB2, HER-2, STAT3, CCND1, MUC1, FGFR3*

## Discussion

This study has revealed that FFPE core biopsies can be used for integrative analysis of gene expression and copy number in breast cancer. The majority of core biopsies from patients could be used for gene expression analysis. Our findings have significant implications for the wider application of FFPE tissue in translational research and clinical use [2, 37, 38].

It is important to compare results from these new platforms with other established methods for molecular marker analysis, such as IHC. When gene expression was compared with IHC for routine markers, concordance was significant (*ER*; *p* = 0.002, *HER-2*; *p* < 0.001, and *PR*; *p* = 0.0002). Very few studies have attempted to compare gene expression profiling with IHC. Gong et al. used gene expression profiles of FF tissue to determine *ESR1* and *HER-2* status among a cohort of 495 breast cancer patients. The data from 195 tumours were used to define the cut-off, and the accuracy of the cut-off was assessed in 300 samples from two further series. This revealed that Spearman’s correlation coefficient ranged from 0.62 to 0.77. The correlation coefficients for *ESR-1* and *HER-2* were 0.77 and 0.81, respectively, for the best-performing probes using the DASL assay in these samples. This is very encouraging, and by using these probes, ER and HER-2 status can be reliably determined.

Abramovitz et al. also showed that there was high concordance between ER, PR and HER-2 IHC and DASL (*p* < 0.01), with significant differences between positive and negative tumours [39]. This degree of agreement is very similar to the extent of concordance observed when *ER* and *HER-2* FISH (fluorescence in situ hybridisation) assessments are carried out on the same samples in different laboratories [40]. These data are of interest as they suggest that DASL is a useful tool for molecular marker analysis.

We identified a high correlation between IHC for ERBB2 and MIP amplification of FFPE breast cancer specimen’s 17q13 loci, with 83% concordance between the two platforms. The concordance was 73% in Andre et al.’s group of 68 patients and 100% in the 61 patients in Pierga et al.’s series. Both these studies used FF DNA, which further confirms the robustness of the MIP assays [41, 42].

Nucleic acid base modifications and cross-linking occur following formalin fixation, rendering intact nucleic acid extraction difficult from these tissues [43]. Many groups have extracted DNA from FFPE tissue blocks and have shown that considerably less DNA can be extracted from FFPE tissues than from FF samples, and this limits its application [44]. Jacobs et al. and Oosting et al. used FFPE DNA from tumour for CNA employing the Affymetrix 500 K array and llumina SNP BeadChip. These platforms require 0.25–1 μg of DNA to generate genome-wide copy number data [45, 46]. The results from these investigations showed that the majority of core biopsy samples contain sufficient DNA for analysis using the Affymetrix 500 K array, but that only 60% of the samples in these studies could be analysed using the Illumina platform. These analyses used FFPE tissue blocks from which to extract DNA, but there is a considerable amount of material available from the initial diagnostic core biopsies in a majority of cases.

The MIP assay is well suited to the analysis of FFPE DNA as it only requires an intact 40-bp sequence and <40 ng of DNA as template [24]. Limitations of other platforms include a requirement for high DNA quantity and quality, and a high output of DNA following amplification, which cannot be achieved using PCR of FFPE samples, Furthermore, many samples exhibit PCR amplification bias due to short template fragment size [47].

Recurrent gains in chromosomes 1q, 8q, 11q, 17q and 20 q and losses in 6q, 8p, 13q and 16q were frequently observed in this study. These identified copy number aberrations were similar to those described in previous studies where genome-wide changes were analysed by chromosomal CGH [48] and aCGH [23, 41, 49–51]. The majority of copy number studies to date have been carried out using FF samples and have employed platforms requiring high DNA input. Most studies have identified 8q, 11q, 17q and 20q as the most frequently gained regions. Andre et al. described gains in 8q (58%) and 1q (55%) and losses in 8p (51%) and 13q (41%) as some of the commonest copy number changes using FF DNA, which concurs with our data, whereas gains in 11q, 17q and 20q were not frequent in their data set [41]. The study by Nessling et al. *is* one of the published studies where locus copy number changes in breast cancer using FFPE tissue were examined. Our data presented here overlap well with their findings. The authors of that study used a matrix-CGH platform containing 422 autosomal markers to profile 31 tumours, and it is encouraging that comparable changes can be identified in FFPE tissue using different platforms, as this suggests that the DNA from such samples is a reliable, readily obtained source for DNA analysis.

Gains in 1q and 8q and losses in 16q were observed in ER-positive cancers, as described by others, while ER-negative tumours were more likely to over-express 17q loci – consistent with the observations of other groups [52].

This study was a feasibility study investigating the role of the integrative analysis of core biopsies for copy number and gene expression profiling and did not aim to discover new molecular markers. Even so, there were several regions in 1q, 8q and 20q that showed alterations in breast cancer with a pCR compared with those without pCR; these differences were not statistically significant. Using 106 fresh tumours, Andre et al. showed that regions in 3q, 6p, 11p, 11q, 18q and 19q were significantly altered in breast cancers showing pCR, compared with non–responders, but this did not reach statistical significance when adjusted for multiple testing (FDR = 0.59) [41].

This study used core biopsy tissue for RNA and DNA extraction and this data confirms that this is a resource that is suitable for molecular marker studies. In a large population-based study, tissue cores from melanoma biopsies were used for profiling using the DASL assay; only 1.4% of the samples failed to yield sufficient RNA using this approach. This is dependent on the age of the sample as older tissue blocks have lower yields and higher level of degradation [53].

Differential expression analysis was aimed at validating the platform further and identifying potential new molecular markers. Differential expression analysis between ER-positive and -negative tumours revealed that all three alternatively-spliced mRNA transcripts of *ESR1* are (with *TFF1)* represented in the top four differentially-expressed genes. This is highly significant as we would expect the main discriminator to be *ESR1* between these cohorts. *TFF1* is also a well-characterised oestrogen-regulated gene and has been shown to be upregulated in ER-positive breast cancers in multiple data sets [54, 55].

A proportion of gene expressions can be accounted for by underlying copy number alterations. Thus, identification of oncogenes that are amplified or lost may lead to the discovery of potential new molecular markers [56]. CNA will help to reveal which amplicons or regions are frequently altered in cancers. Subsequently, genes at these loci can be studied in more detail to characterise potential oncogenes or tumour suppressor genes. In addition, a high correlation was observed between copy number and gene expression in regions commonly amplified in breast cancer, such as 20q13 (*B-MYB*), 17q (*HER-2*) and 11q (*CCND1*).

There was a good correlation between copy number and expression among the pCRs for several genes including *HER-2*, *CCND1, TNFAIP1* and *JUND*. This is a well-established relationship as many studies have shown that *HER-2* positivity is associated with higher response rates to NAC [57]. Penault-Llorca et al. observed that *CCND1* positivity with IHC was increased following neoadjuvant chemotherapy, but they were unable to show a correlation with response to chemotherapy [58]. *CCND1* is a frequently amplified gene in breast cancer and has been previously shown to be associated with improved disease-free and overall survival.

The genes assessed as part of the DASL cancer panel assay consisted of 502 genes derived from various published data sets where gene selection is biased towards haematological malignancies. This is not an ideal setting for ‘class discovery’ in breast cancer but represents a useful platform for an initial pilot study [59]. The unbiased, DASL WG assay – where all transcripts are assessed – is a superior platform, but was not available at the start of this study. This is a ‘bottom-up’ model for molecular marker studies as it only assesses a limited number of markers, although this approach has been successfully used in other studies [60]. For example, a similar approach was adopted by Paik et al. for deriving their Recurrence Score™, where they used an initial list of 250 genes from published studies; this was then used to arrive at their final selection of 16 genes [38]. A recent consensus statement on FFPE analysis suggested initially using a targeted pilot project to assess the robustness of the platform.

In our study, only a relatively small number of samples were investigated, and therefore it has a constraining power in detecting genes of significant effect, as shown in many other studies of similar size [61–63]. Nevertheless, ours was one of the first analyses of an FFPE platform to be carried out for breast cancer, to assess the platform and robustness compared with an IHC approach, using either DASL or any other technology for gene expression profiling employing FFPE tissue.

The results obtained using the DASL assay are comparable with those for IHC and the DASL assay has the potential to assess large numbers of markers, hence enabling large numbers of samples to be evaluated for potential molecular markers. This is particularly valuable in cancers where there is limited availability of fresh tissue. In our study, core biopsy samples were successfully used for CNA using an MIP assay. This shows that both the DASL and the MIP assays provide a valuable tool for molecular marker studies in cancer.

## Conclusion

Integrated genomic and transcriptomic analysis of FFPE samples will allow rapid progress in molecular marker identification in cancer research. This is one of the first studies, to have shown the use of FFPE samples to assess gene expression and copy number analysis. The expression profiles from FFPE samples correlated with those from FF samples. Therefore, FFPE samples can be more readily used for translational research in the future.

## Additional files

Additional file 1: Clinical Data - The Sample ID and the Nature of samples (C-cancer or N-Normal), Tumor grade (1, 2, or 3, N/A) and oestrogen Receptor Status (1-positive, 2-negative or N/A- not available). (TXT 1 kb)

Additional file 2: Oestrogen Receptor data. The Oestrogen receptor SNP’s and their effect on probe expression (ESR- SNPs). Box plot of the expression in each of the three probes of ESR1 against each SNP (no significant difference on ANOVA). (PDF 16 kb)

Additional file 3: Molecular Inversion Probe Copy Number data. The data including probe signal, probe position, call rate, copy number relative standard deviation, Copy Number and Allelic ratio. (TXT 84778 kb)

Additional file 4: DASL Assay sample sheet (Assay ID 4139683017). The sample sheet containing the sample ID, replicates, array position. (CSV 5 kb)

Additional file 5: DASL Assay sample sheet (Assay ID 4139683008). The sheet sample containing the sample ID, replicates, array position. (CSV 4 kb)

Additional file 6: DASL Data from Assay ID 4139683017. (ZIP 4853 kb)

Additional file 7: DASL Data from Assay ID 4139683008. (ZIP 2739 kb)

## Abbreviations

AUC: Area Under the Curve
CNA: Copy Number Analysis
CGH: Comparative Genomic Hybridisation
DASL: cDNA-Mediated, Annealing, Selection, Extension and Ligation Assay
DNA: Deoxyribonucleic acid
ER: Oestrogen Receptor Protein
ESR: Oestrogen Receptor Gene
FFPE: Formalin Fixed Paraffin Embedded
HER-2: Human Epidermal Growth Factor Receptor Type 2 Protein
IHC: Immunohistochemistry
MIP: Molecular Inversion Probes
NAC: Neoadjuvant Chemotherapy
PCR: Polymerase Chain Reaction
PGR: Progesterone Receptor Gene
PR: Progesterone Receptor Protein
RNA: Ribonucleic Acid
SNP: Single Nucleotide Polymorphism
