# Supplementary material for: Integrative analysis of copy number and gene expression in breast cancer using formalin-fixed paraffin-embedded core biopsy tissue: a feasibility study
Source: BMC Genomics. 2017 Jul 11;18:526. doi: 10.1186/s12864-017-3867-3 (PMC5506605; doi:10.1186/s12864-017-3867-3)
Supplement: Supplementary file 2 — Oestrogen Receptor data. The Oestrogen receptor SNP’s and their effect on probe expression (ESR- SNPs). Box plot of the expression in each of the three probes of ESR1 against each SNP (no significant difference on ANOVA). (PDF 16 kb) [file 12864_2017_3867_MOESM2_ESM.pdf]

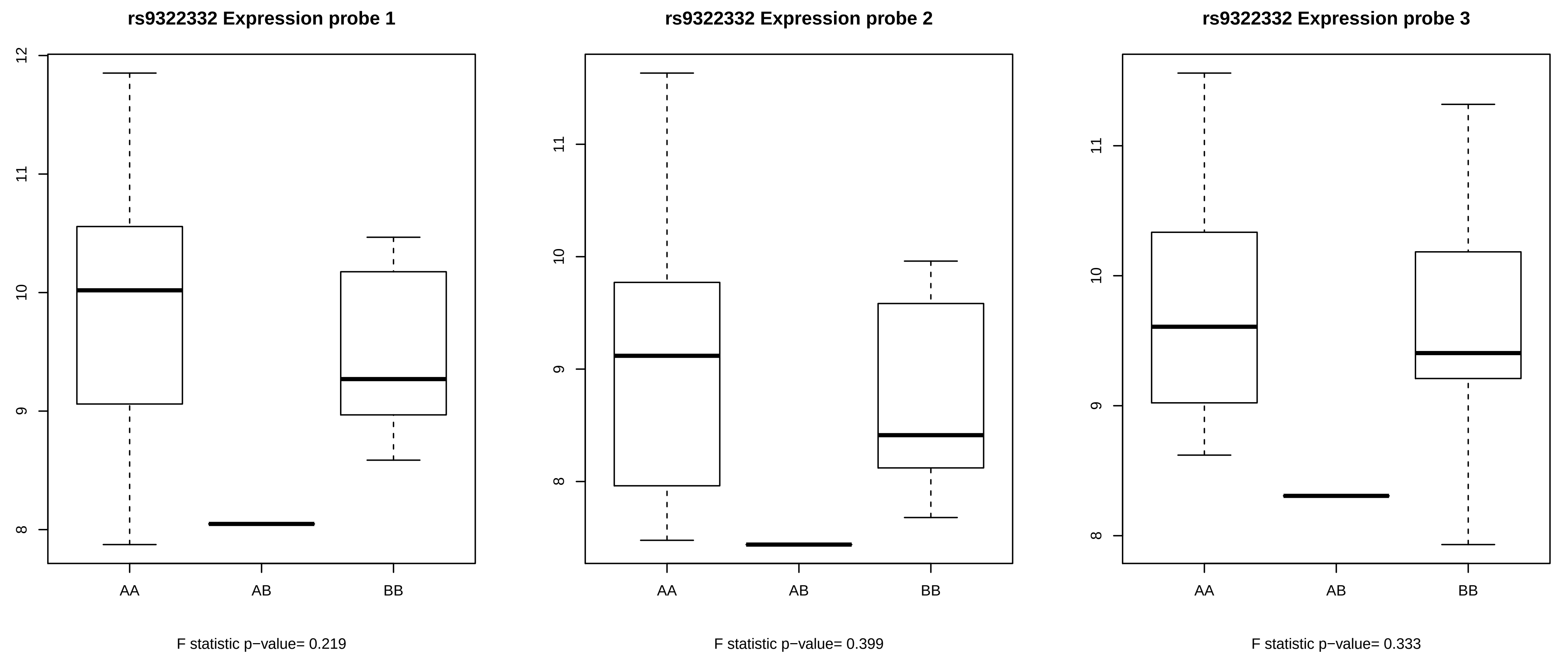
rs9322332 Expression probe 1
12
11
10
9
8
AA
AB
BB
F statistic p−value= 0.219
rs9322332 Expression probe 2
11
10
9
8
AA
AB
BB
F statistic p−value= 0.399
rs9322332 Expression probe 3
11
10
9
8
AA
AB
BB
F statistic p−value= 0.333

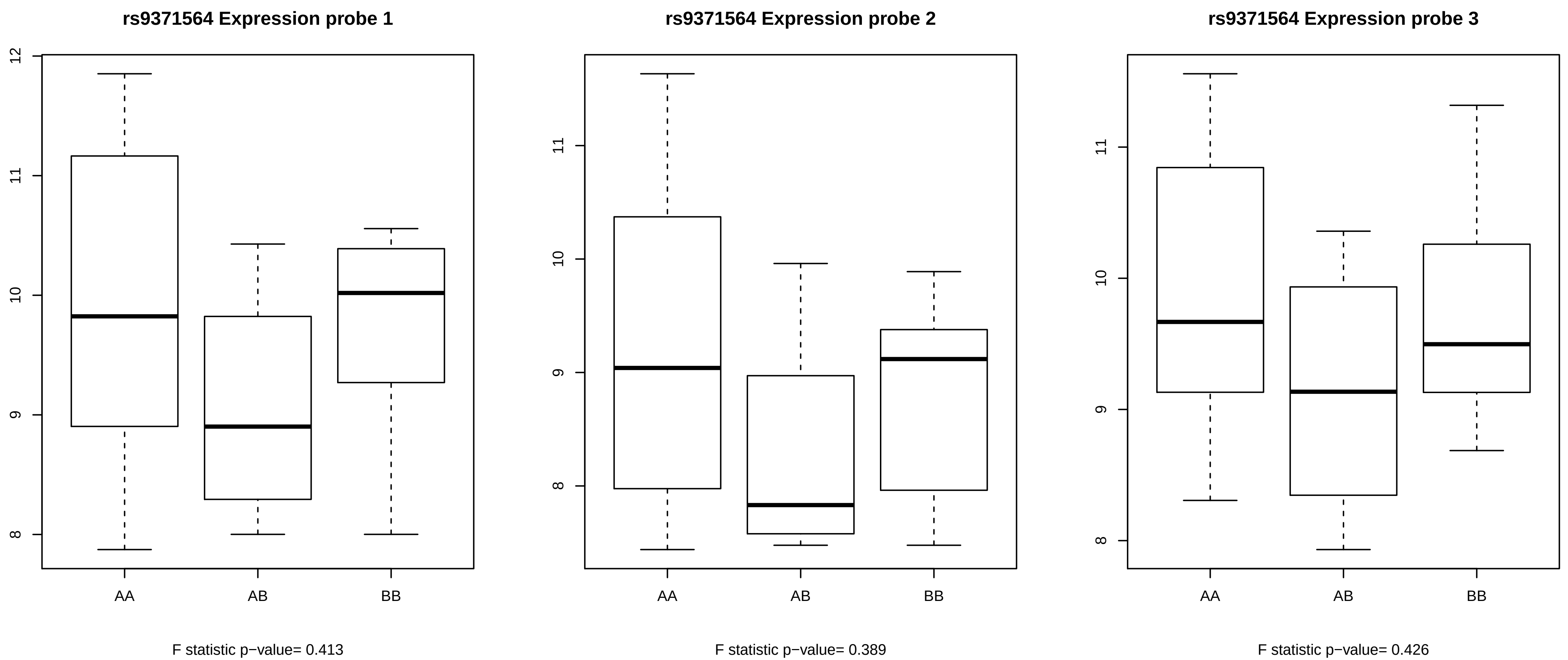
rs9371564 Expression probe 1
rs9371564 Expression probe 2
rs9371564 Expression probe 3
12
11
10
9
8
AA
AB
BB
F statistic p−value= 0.413
F statistic p−value= 0.389
F statistic p−value= 0.426

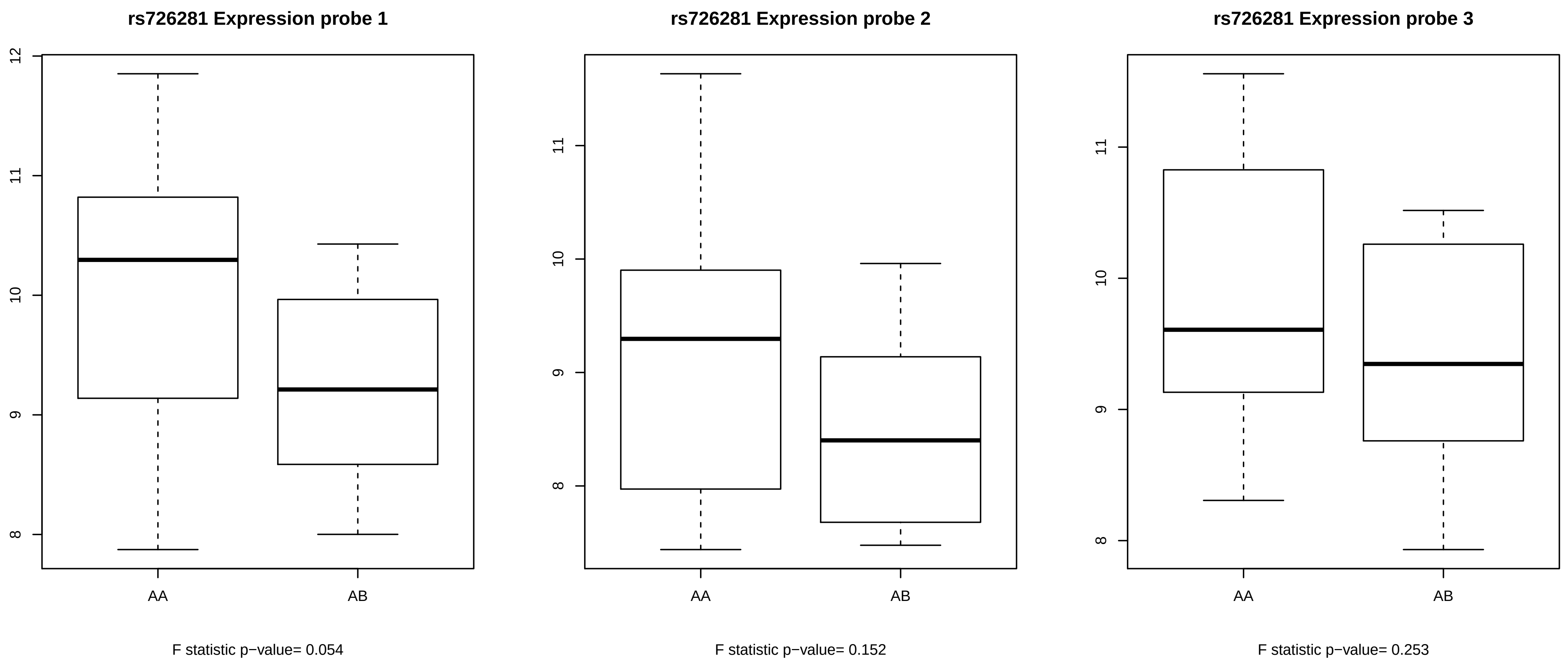

rs726281 Expression probe 1
12
11
10
9
8
AA
AB
F statistic p−value= 0.054
rs726281 Expression probe 2
11
10
9
8
AA
AB
F statistic p−value= 0.152
rs726281 Expression probe 3
11
10
9
8
AA
AB
F statistic p−value= 0.253

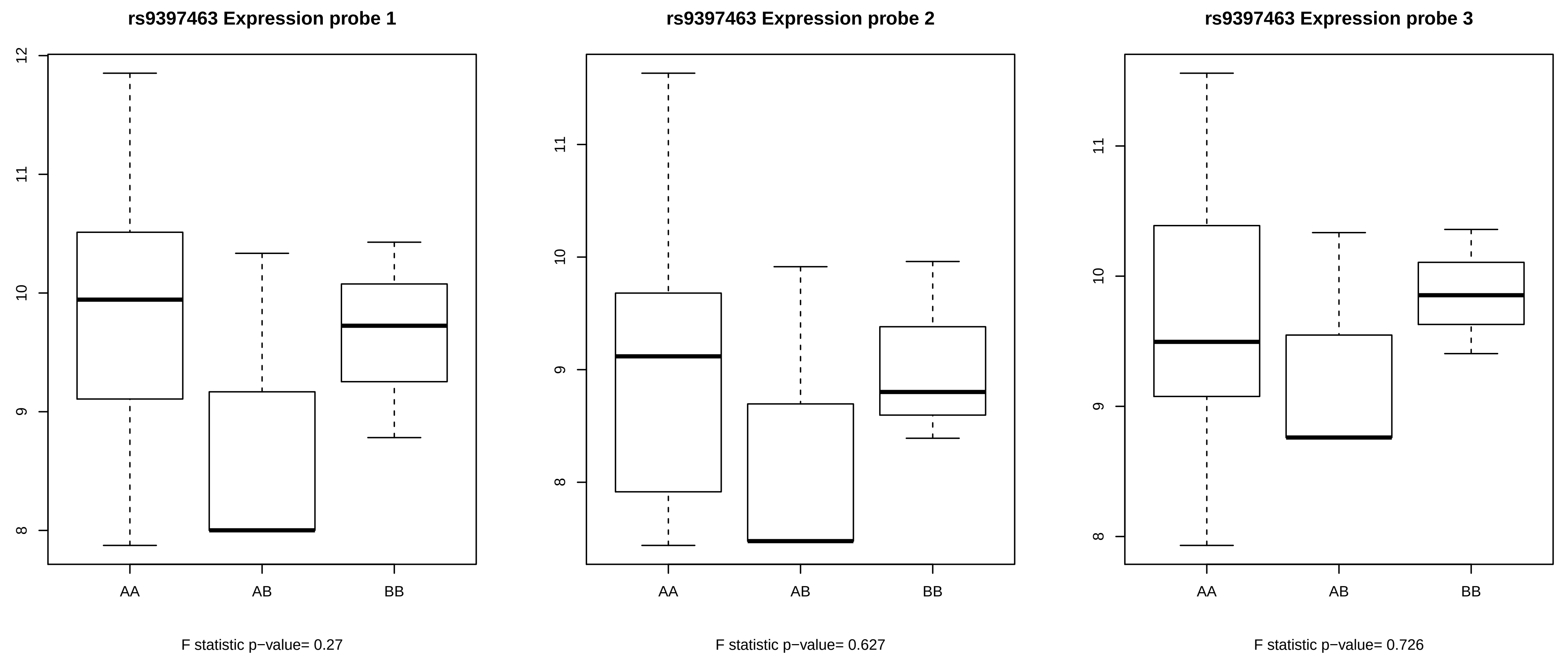
rs9397463 Expression probe 1
rs9397463 Expression probe 2
rs9397463 Expression probe 3
12
11
10
9
8
AA
AB
BB
F statistic p−value= 0.27
F statistic p−value= 0.627
F statistic p−value= 0.726

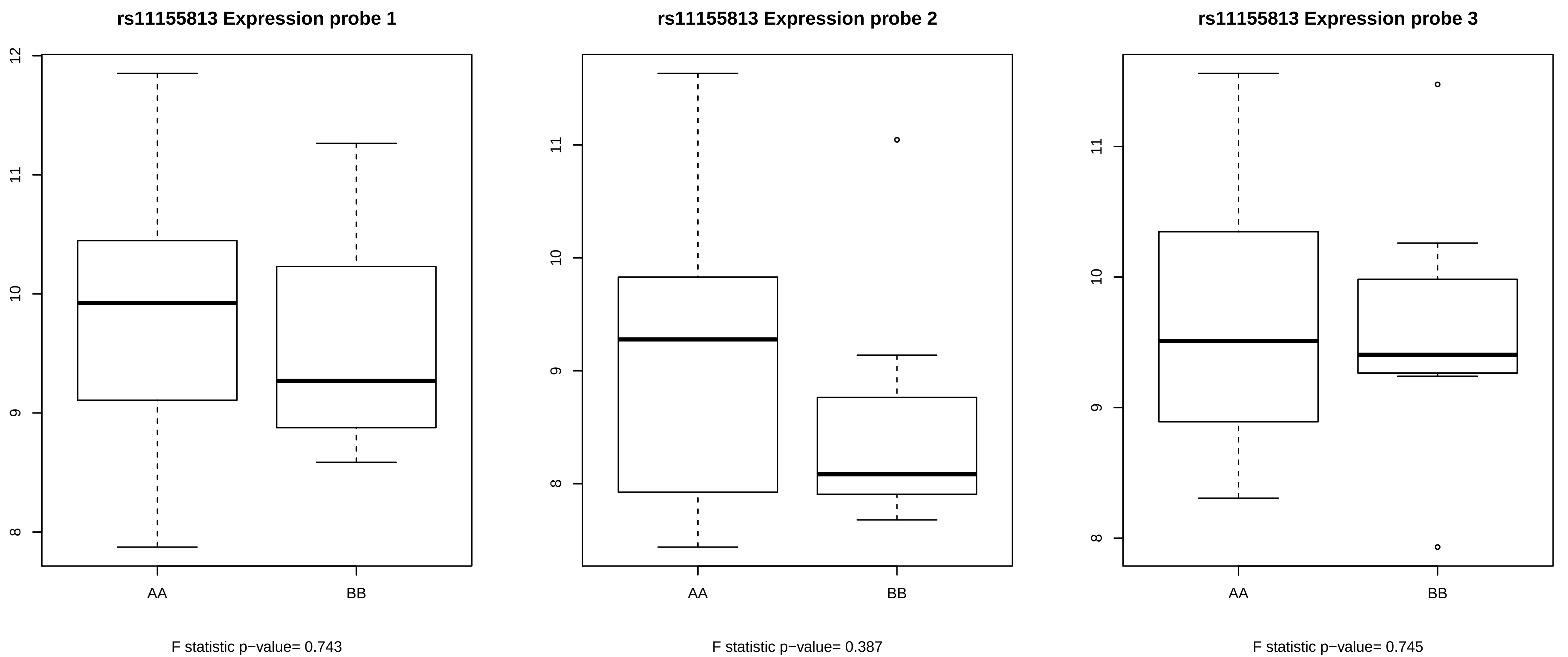
rs11155813 Expression probe 1
rs11155813 Expression probe 2
rs11155813 Expression probe 3
AA
BB
F statistic p−value= 0.743
F statistic p−value= 0.387
F statistic p−value= 0.745

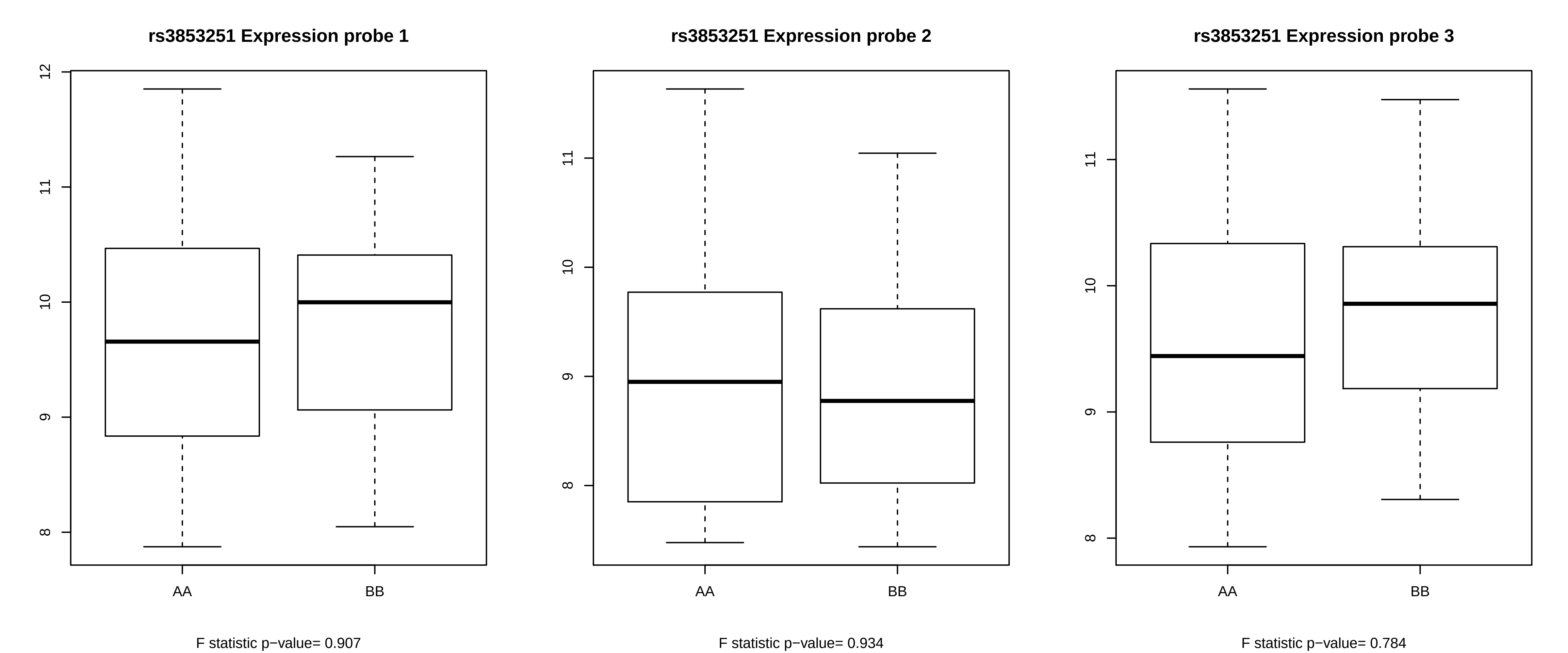
rs3853251 Expression probe 1
12
11
10
9
8
AA
BB
F statistic p−value= 0.907
rs3853251 Expression probe 2
11
10
9
8
AA
BB
F statistic p−value= 0.934
rs3853251 Expression probe 3
11
10
9
8
AA
BB
F statistic p−value= 0.784

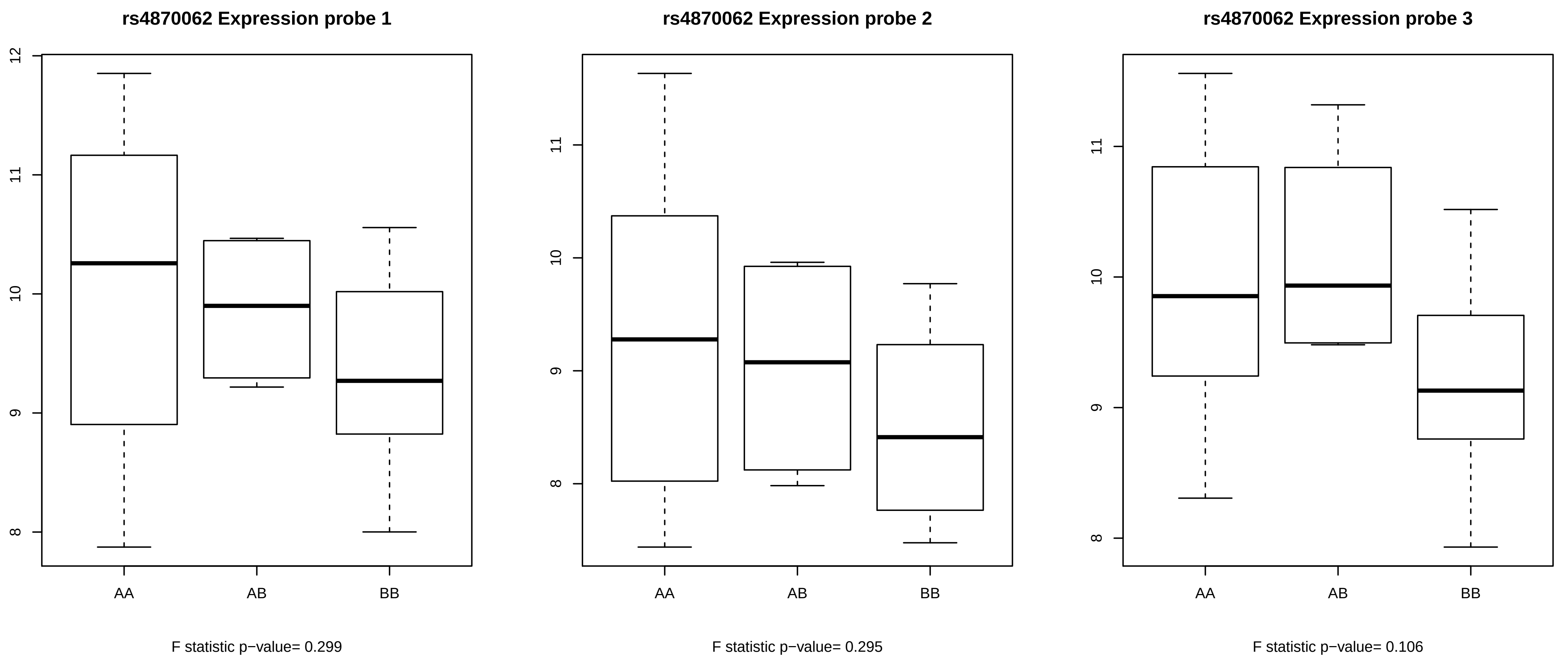
rs4870062 Expression probe 1
12
11
10
9
8
AA
AB
BB
F statistic p−value= 0.299
rs4870062 Expression probe 2
11
10
9
8
AA
AB
BB
F statistic p−value= 0.295
rs4870062 Expression probe 3
11
10
9
8
AA
AB
BB
F statistic p−value= 0.106

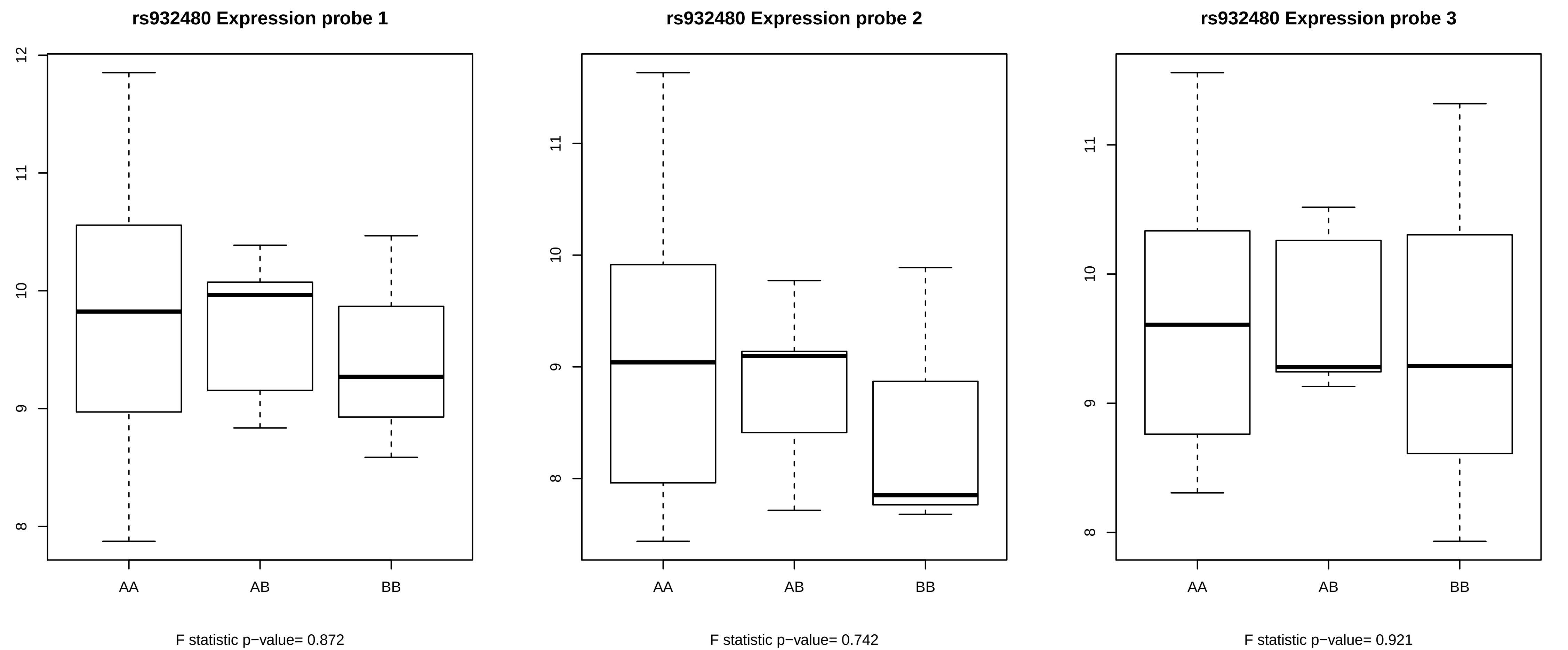
rs932480 Expression probe 1
rs932480 Expression probe 2
rs932480 Expression probe 3
AA
AB
BB
F statistic p−value= 0.872
F statistic p−value= 0.742
F statistic p−value= 0.921
