# Supplementary figures and images for: Integrative analysis of copy number and gene expression in breast cancer using formalin-fixed paraffin-embedded core biopsy tissue: a feasibility study
Source: BMC Genomics. 2017 Jul 11;18:526. doi: 10.1186/s12864-017-3867-3 (PMC5506605; doi:10.1186/s12864-017-3867-3)

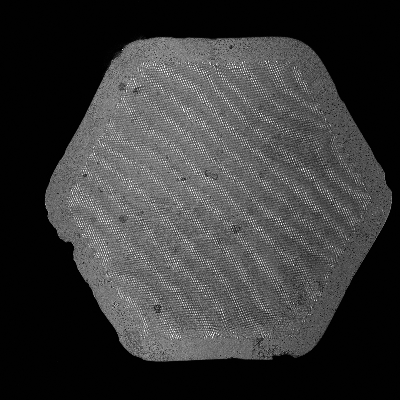

Supplement: Supplementary file 6 — DASL Data from Assay ID 4139683017. (ZIP 4853 kb) [file 12864_2017_3867_MOESM6_ESM.zip › 4139683017/4139683017_fiducial #1_Red_center.tif]
